# “Engagement Is Not One‐Dimensional”: The Multiple Roles of Patient Educators With Physical Disabilities in Family Medicine

**DOI:** 10.1111/hex.70851

**Published:** 2026-08-26

**Authors:** Vivien Min Er Lee, Laurie J. Goldsmith, Anna Szücs, V Vien Lee, Jun Cong Goh, Jeffrey Jiang, Nicola Ngiam, José M. Valderas, Victor Weng Keong Loh

**Affiliations:** ^1^ Division of Family Medicine, Department of Medicine, Yong Loo Lin School of Medicine National University of Singapore Singapore Singapore; ^2^ National University Polyclinics Singapore Singapore; ^3^ Faculty of Behavioural and Movement Sciences Vrije Universiteit Amsterdam Amsterdam Netherlands; ^4^ Department of Post‐Acute & Continuing Care, Jurong Community Hospital National University Health System Singapore Singapore; ^5^ Centre for Healthcare Simulation National University of Singapore Singapore Singapore

**Keywords:** communications curriculum, family medicine, patient educator, patient voice, persons with disabilities

## Abstract

**Introduction:**

Patient interaction has long been central to medical training. In recent years, involving patients as educators and bringing their lived experiences of illness or disability to the classroom has been valued in clinical education. These “experts by experience” enrich learning by offering authentic perspectives that foster empathy, challenge assumptions, and promote person‐centred care. To our knowledge, there is no existing research on the experiences of persons with disabilities (PWD) serving as patient educators.

**Methods:**

Semi‐structured in‐depth interviews conducted with seven patient educators with physical disabilities (all PWD patient educators who consented to the study) about their experiences teaching in an undergraduate medical student workshop on communication with PWD. Reflexive thematic analysis was used for analysis.

**Results:**

Illustrating that “engagement is not one‐dimensional,” participants described engaging students through three overlapping and dynamic roles: patient, educator, and advocate. Four distinct types of patient educator were identified: Type A (Patient role is more predominant than the Educator role), Type B (Patient and Educator roles are equally important with less emphasis on the Advocate role), Type C (Patient, Educator, and Advocate roles are all equally important), and Type D (Patient and Advocate roles are equally important with less emphasis on the Educator role). Patient educators who demonstrated stronger integration of the educator role (Types B and C) were more confident, pedagogically effective, and aligned with curricular aims than those in Type A, whereas those whose engagement was primarily advocacy‐oriented (Type D) occasionally diverged from educational goals.

**Conclusions:**

This study highlights the multidimensional nature of patient educator roles and identifies the strengths and limitations associated with different engagement orientations. Recognising and supporting these intersecting identities can inform recruitment and facilitation practices. Structured training and guided reflection may help patient educators balance personal narratives and advocacy aims with educational objectives, thereby optimising learner outcomes and strengthening the meaningful inclusion of PWD voices in medical education.

**Patient or Public Contribution:**

This study focuses on the perspectives of PWD as educators in an undergraduate medical student workshop on communication with PWD. This workshop was co‐created with patient educators with disabilities.

**Abbreviation**
PWD: persons with disabilities

## 1 Introduction

Medical training has long emphasised patient contact as a cornerstone of clinical education. This has evolved to include roles for patient educators, that is individuals who contribute to medical training by sharing their lived experiences of illness or disability. Described as “experts of their experience” [1, 2], patient educators add a rich, human perspective to clinical teaching that transcends textbook knowledge and didactic instruction [1, 3, 4, 5]. Through storytelling, dialogue, and authentic interaction, they provide learners with insights into the realities of illness, disability, and healthcare navigation that are often overlooked in conventional curricula. Among the many benefits, engaging with patient educators has been shown to foster empathy, critical thinking, and awareness of social justice issues in learners [6]. These interactions challenge assumptions, promote inclusive attitudes, and sensitise future healthcare professionals to the lived realities of patients.

Involving patient educators in medical education around care of persons with disabilities (PWD) has been deemed essential [7]. Having PWD as patient educators enables holistic and richer perspectives in medical education [8] as patient educators' years of experience dealing with their own complex medical conditions and disabilities make them an important resource for the students [9]. Direct contact with PWD can play a powerful role in shaping attitudes and clinical communication skills that are respectful, person‐centred, and equity‐driven [10, 11, 12]. However, despite this need for direct contact, medical students receive limited, if any, education about PWD and how to communicate and provide care for PWD. This lack of awareness and training exacerbates healthcare disparities experienced by PWD [13].

There is under‐representation of teachers with disabilities in research and in the teaching profession [14]. While research exists focusing on the lived experience and training of teachers with disabilities and staff and students' perspectives about teachers with disabilities, none of these studies include perspectives of teachers with disabilities in medical schools [14]. To the best of our knowledge, no research exists on the perspectives of patient educators who themselves live with disabilities. The role of PWD patient educators is likely complex, integrating their identity as a PWD with other personal and professional identities. Disability has been increasingly viewed as an identity in itself [15], yet research is scarce on how it integrates with a person's other identities [16]. Understanding PWD patient educators' motivations, challenges, and evolving roles within educational contexts through this lens appears a useful way to reinforce their ability to engage learners, and their alignment with workshop goals by refining recruitment, selection, and training of future PWD patient educators with disabilities. Developing this understanding can help ensure that PWD patient educator involvement is both meaningful and effective in enriching medical education.

This study thus aimed to explore how PWD experience their role as patient educators in medical education.

## 2 Methods

### 2.1 Setting

In 2022, the National University of Singapore introduced a “Communication with Persons with Disabilities” workshop into the third‐year undergraduate curriculum for medical students during their family medicine rotation [12]. The workshop was co‐created with PWD and includes PWD as patient educators. The learning objectives are listed in Box 1. This study took place across the first and second year of the workshop operation. Each workshop was attended by 70–80 medical students and lasted 4 h.

Box 1 **Learning objectives of the Communicating with PWD workshop.**

1

1. List common societal assumptions about PWD.
2. Describe how the experience of disability results from a confluence of individual, provider and societal/structural factors.
3. List tips for communicating respectfully with PWD.
4. Reflect on our roles as future healthcare providers and how we may respectfully engage with PWD in our clinical interactions

Each workshop started with an hour of plenary session highlighting key concepts, followed by three different stations with PWD patient educators that depicted a different disability (vision, hearing, and mobility) each. There were two circuits, with a total of six stations. Observed by [12] to [14] peers in a fishbowl format [17], each station is comprised of a 10‐min simulated consultation between a student volunteer and a PWD patient educator who enacted a scripted role consistent with his/her actual disability. Feedback was provided by peers, a tutor, and the PWD, followed by a facilitated 30‐min‐long discussion where students can then ask the PWD questions. Typical questions posed to the PWD in past workshops had included how they became a PWD, how they experienced the healthcare system, their social encounters, and how they coped in daily life. The students were given reflection sheets to pen their reflections throughout the workshop. After all groups have rotated through the three stations, the workshop ends with a 45‐min session of sharing student reflections and learning points. The workshop was shown to increase medical students' attitudes towards PWD favourably [11].

PWD with vision, hearing and mobility disabilities were recruited to participate as patient educators in the “Communication with Persons with Disabilities” workshop through the Standardised Patient programme at the Centre for Healthcare Simulation in National University of Singapore. PWD recruitment began with referrals from medical students and social service agencies. Candidates' motivation to participate and fit for the role were assessed by an online application form followed by a face‐to‐face interview. Selected individuals attended a half‐day course, which provided information on the operational aspects of the programme. This course also provided training on the role of a simulated patient and the essentials of giving feedback. The case scenarios for the PWD were developed to allow medical students to understand the challenges faced by PWD in their encounters with healthcare. The PWD patient educators were paid at the same hourly rate as Standardised Patients, namely at 25 SGD/hour.

### 2.2 Study Design and Procedure

All 14 patient educators with disabilities who taught in the workshop between June 2022 and October 2023 were sent email invitations to participate in the study. Consenting to participate in the study consisted of providing written informed consent and completing a short survey about selected demographics (age, gender, ethnicity), duration of their patient educator activity, their type of disability, and communication preferences during the interview (e.g., live transcription or sign language interpretation for persons with hearing loss).

A semi‐structured interview guide was developed by the study team and further adapted throughout data collection (see Supporting Information: Material, S1, p.4 for the initial version). The initial interview guide included three main sections: participants' experience as patient educators, their perspectives on how the PWD curriculum may affect students' humanistic medical professional identity, and the strengths and weaknesses of the workshop. After the first interview, the interviewer (VVL) and lead author (VLME) felt that the most valuable and interesting insights were the patient educators' experience and their role as educators, and the interview guide was revised to focus and expand on this topic (see Supporting Information: Material, S1, p.5 and 6 for the iterative version).

Audio‐recorded, hour‐long, semi‐structured individual interviews were conducted in‐person or via Zoom by an experienced interviewer (VVL, a female post‐doctoral fellow with psychology training) who had no prior contact with participants. Reflexive notes were taken by the interviewer at the end of each interview. Participants were not compensated for study participation. All procedures were approved by the Institutional Review Board of the National University of Singapore (NUS‐IRB‐2023‐154).

### 2.3 Data Analysis

All recordings were de‐identified and transcribed verbatim. A combination of Microsoft Word and Microsoft Excel was utilised for coding and categorisation of themes.

Analysis consisted of reflexive thematic analysis [18]. Analysis was primarily inductive, allowing construction of themes through engagement with patient educators' accounts rather than being organised around a pre‐existing theoretical framework. The lead author (VLME, a family physician) coded all the transcripts, while meeting regularly with other research team members with qualitative expertise (LJG, a health services researcher, and AS, a mental health researcher) to develop the coding framework, and identify initial themes and an organising thematic narrative. All transcripts were read and analytically annotated by the second analyst (LJG) early in this iterative analysis process to augment the lead author's initial line‐by‐line coding. The three primary analysts agreed on the typology descriptions and typology characterisation of each study participant with any disagreement resolved through discussion. The final set of themes, accompanying narrative, and typologies were refined through discussions with the rest of the study team whose members were from multiple disciplines (family medicine, medical education, psychology, psychiatry, clinical healthcare simulation, health services research). Having multiple team members involved in analysis in multiple ways ensured analytic rigour.

In the following section, quotations are reproduced verbatim with hesitation sounds and repetitions deleted. Deleted elements are indicated “…” and clarifying comments were added within square brackets, “[]”, where needed. Readers accustomed to British or American English may find Singlish (a colloquial English‐based creole spoken in Singapore that incorporates elements of Chinese and Malay) expressions curious at first. Such expressions were paraphrased in comments in places where they could impact understanding.

## 3 Results

### 3.1 Participant Characteristics

Seven of the 14 patient educators participated in the study. Non‐participating patient educators were not required to provide a reason for not participating and no reason was spontaneously given. Study participants had between 1 and 3 years of experience as a patient educator and reported their disability as mobility impairment, vision impairment, or hearing loss. They varied in age from 34 to 64 years, and the majority were male and of Chinese ethnicity (Table 1).

**Table 1:** Participant characteristics (*n* = 7).

Characteristics		*n*
Years of experience as patient educator	Less than 1 year	1
	1 year to less than 2 years	4
	2 years to less than 3 years	2
Type of disability	Vision	4
	Hearing	1
	Mobility	2
Age	30–39 years	1
	40–49 years	1
	50–59 years	3
	60–69 years	2
Gender	Female	3
	Male	4
Ethnicity	Chinese	4
	Malay	2
	Eurasian	1

### 3.2 Engagement in Three Different Roles (Patient, Educator, Advocate)

**Figure 1:**
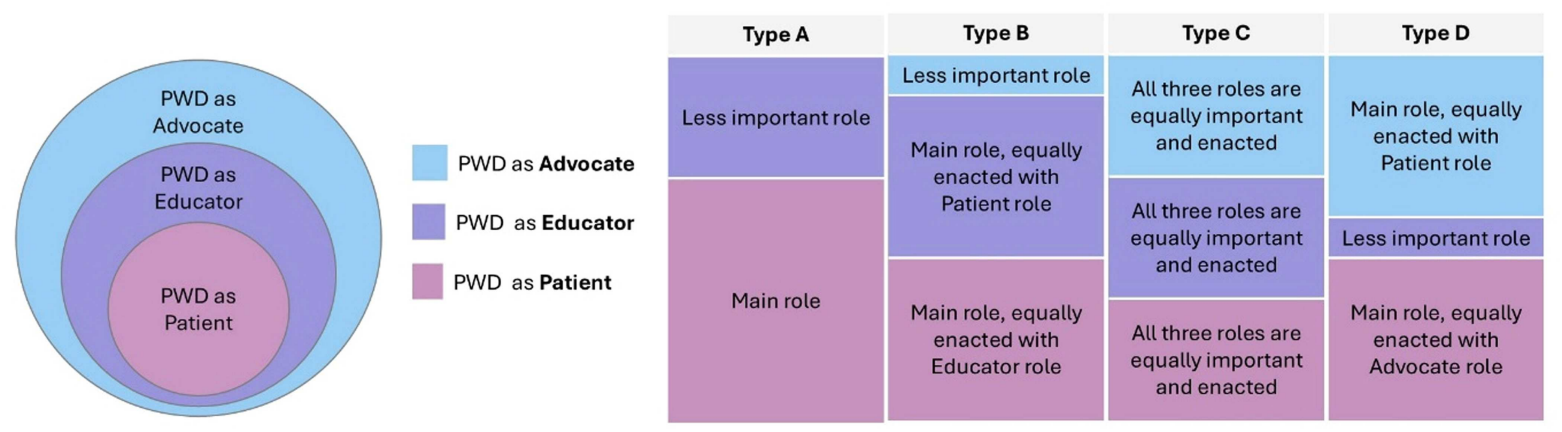
Roles played by PWD patient educators. *Note:* The left panel illustrates how the three roles, namely patient, educator, and advocate relate to each other, going from the most individual role (innermost layer) to the most societal one (outermost layer). The right panel illustrates the proportion at which each of these three roles is present in the different types of patient educators identified by the study. The height of rectangles indicates the extent a role is important for a given type. The absence of a rectangle means that a role is not part of that type's identity. PWD, persons with disabilities..

Patient educators with disabilities participating in the medical school curriculum described how they interacted with the students in more than one way (“*the engagement is not one‐dimensional*”). They not only brought to the table their personal experiences as *patients*, they also operated as *educators* through teaching practical knowledge and tips beyond medical textbooks. Some patient educators additionally functioned as *advocates for PWD* to promote inclusivity in healthcare and more broadly within society. As illustrated in Figure 1, these roles varied by focus. When patient educators provided reflections on their healthcare experience as PWD, the focus was on them as individuals. When they guided medical students in their interactions, they operated as educators. For a subset of patient educators who also functioned as advocates in the classroom, the focus at times further shifted to society at large.

patient educators simultaneously assumed these multiple roles. These roles were fluid rather than fixed and each individual varied their emphasis on these roles. While all patient educators consistently identified first and foremost as patients, the relative weight they gave to their additional roles as educators or advocates varied. This diversity of identities significantly shaped patient educators' teaching approach and confidence.

Four distinct types of patient educator were identified: Type A (*Patient role is more predominant than the Educator role*), Type B (*Patient and Educator roles are equally important with less emphasis on the Advocate role)*, Type C (*Patient, Educator, and Advocate roles are equally important*), and Type D (*Patient and Advocate roles are equally important with less emphasis on the Educator role*) (Figure 1). These identities reflected different orientations toward personal storytelling, teaching practices, and advocacy priorities, as described in more detail below. Illustrative quotes for each type are provided in Table 2.

**Table 2:** Illustrative Quotes.

**Type A Patient Educator (Patient > Educator)**
***Lived‐in experience as patient is most natural***
"*[The workshop] is quite structured, very rigid I should say … they give a scenario, and you just have to follow it through.”*
*“I would just treat myself as a patient in front of them. So just to be as real as possible even though the scenario could be not what I am…”*
*“When the students start to ask me questions about how I lost my hearing, and my life and my challenges [living with hearing loss] … I share with them.”*
***Being a teacher is a novel experience***
*“When I did the first [teaching session]… I was nervous but [after the session started]… I think [these students] were more nervous looking than me. So in the beginning … I really didn't know what to expect… then later on I realised … it's a learning journey for these students, so I need to let them know … what is the right way and what is not the right way [to communicate with PWD]. After listening to what I had to say, I guess they are more confident of [communicating with PWD] After that first time, I became less nervous and I could actually share more things [with the students].”*
*“… practice makes perfect. [Initially] I was rather nervous and tense, and I do not know how to express … the last few sessions are much easier … I'm able to bring up some of my experiences in a more natural way…”*
**Type B Patient Educator (Patient = Educator > Advocate)**
***Demonstrates strong commitment to teaching effectiveness***
*“I would read up … to contribute to the case studies. so at least there's a variety of the things I can share, I mean in the event that [the students] do face those stuff, at least they are well prepared, right?”*
*“I always share tips from my own experience, but I can also share tips from my friends' experiences. Because I am totally blind, my experiences will be different from a friend or even my husband who is partially blind.”*
***Engaging and giving constructive feedback to students to maximise their learning***
"*… Even people with the same disabilities have different needs. So for us [patient educators], [we] can also learn [from someone with a different disability] … so next time I can tell someone else, “okay, if you want to interact with a person who's deaf…you shouldn't do this … you should do this. This is a better way to communicate with them.”*
*“Because it's a learning journey for these students, so I need to let them know … what is the right way and what is not the right way.”*
*“I will say number 1 is patience. And number 2 is willingness to help and to educate. And number 3 is to not be shy to share with them in the event they make mistakes, because by sharing their mistakes … in a very polite way … that's the only way for us to learn.”*
**Type C Patient Educator (Patient = Educator = Advocate)**
***Balancing the roles of patient, educator and advocate***
*“There are so many layers as to how we are able to reach out to them as an educator, you know? Our personal stories, as well as that of how the community [with disability] actually thinks or functions*.”
*“I was more than happy to help [as a patient educator] because I feel this is what I should do. Because doctor gave me this cochlear implant… so I have to give back. I simply have to because of the people that come after me, and because the deaf community don't have a voice … I may not live to see the day when every healthcare service provider has one sign language interpreter, has someone who can understand and able to communicate with people with hearing loss.”*
*“As a patient educator, I feel that I am able to impart the life experience … [for] the fact that you want to see this generation go beyond the sympathetic charitable working style, but to a more empathetic one … [making] them feel more human.”*
*“So I mean, although I said I want to have more leeway [in the workshop], but then again there must be some limits to it … [to be] within the [learning objectives]. As educators, I cannot say it's free for all. I understand that.”*
*“I do that because I would [like to] create awareness for people like you [PWD]. If nobody wants to step out and create awareness … then healthcare service provider, especially students, [they have] no awareness … You cannot find this in your books…. The [medical] books don't teach you. People have to want to come out and share with you. First I will want to create awareness like this.”*
**Type D Patient Educator (Patient = Advocate > Educator)**

Views this workshop mainly as an advocacy platform
*“In the last 4 years I have been doing a lot of rebuilding. I have managed to get a job, I have managed to become part of a community of persons with disability, but we like to call ourselves persons with determination. I advocate for both disability…I advocate very strongly for inclusion and mental health.”*
*“You know I have been doing this kind of thing… Everybody asked me. I have appeared in many videos [provides local and international list] … I have appeared in [names Singapore newspapers].”*
*“I think the one thing that I feel [curriculum developers] should do is to have a lot more awareness. I am really heavy on LinkedIn and I see a lot of other stories going about. I think running a story behind the patient educators… The programme has been a little low key, you know? You don't really see much of that information out in the public. I believe it will make a big difference … from a public relations angle that [curriculum developers] has piloted this programme and these are the profiles of the individuals on the programme … these are the PWDs themselves, their journey…”*
*“Changing their attitude is a little bit difficult for me to say because it's their individual attitude. But giving them that extra piece of knowledge to know how to handle the disabled people may be possible… If you give them a little bit of disability awareness and understanding, it may help them a lot more. My motivation is always that if these people get something, I get something. Because if I help them, they will help me next time or they will help other people who are visually impaired next time. So I will be happy … the satisfaction is when you talk to these people and make them aware of it.”*
***Prioritising personal agenda over learning goals***
*“You know it can be just normal interaction and then we see where it goes … but then again, of course this may not be what the professors want, [because] put it this way, it's a non‐controlled thing. Ok, I understand you need to have control and you need to know what kind of things to impart to the student. Therefore, the thing becomes a little bit more rigid…If you just let everything be up to me to say and do, then a lot of things can happen.”*
***Sharing inaccurate or incomplete beliefs about healthcare***
*“[The scenarios are] unrealistic … I honestly think at the end of the day, it is not the medical team that actually wrote the script People who see family doctor will not be PWD. PWD will be the ones who go into the emergency departments and polyclinics [Singapore's public primary care clinics], but they won't be the ones who go to the family doctors, and if they do, it will be for very non‐emergency things, e.g., I have a fever, I have a flu…”*

### 3.3 Type A: Patient > Educator

#### 3.3.1 Patient Role Is More Predominant Than the Educator Role

Patient educators whose primary role centred on their lived experience as patients approached teaching as a secondary responsibility. These patient educators identified as being hired specifically for their disability experiences and performing as simulated patients reinforced the dominance of their patient identity. Their focus was the authentic sharing of personal stories, challenges in healthcare encounters, and the range of accommodations and needs associated with disability. They perceived this authenticity as highly impactful on students, noting visible shifts in students' level of empathy and understanding during interactions.

The patient role came most naturally and easily for these patient educators. Some described difficulty when expected to portray simulated cases that did not align with their own health history. This could create tension between their authentic patient identity and the expectations of simulation‐based teaching.

For some patient educators in Type A, being a teacher was a novel experience. Personal anxieties had to be overcome to share personal stories in front of an audience. However, realising that students were even more nervous than them attenuated their nervousness. Over time, these patient educators gained confidence with sharing.

### 3.4 Type B: Patient = Educator > Advocate

Patient and Educator roles are equally important with less emphasis on the Advocate role

These patient educators had well‐integrated patient and educator roles, both of which were prioritised over their advocacy role. These individuals demonstrated strong commitment to teaching effectiveness. They described deliberate efforts to learn instructional strategies, refine communication, and provide structured feedback that supported students' clinical skill development.

Teaching and speaking in front of an audience was not new for this group. Yet even these more experienced patient educators reported continued self‐learning and self‐reflection on what could be done better to improve their teaching. As they were given more opportunities to teach, Type B patient educators grew further into their role as educators They embraced actively learning from feedback from students and from other patient educators and working well with the tutors who helped to facilitate the sessions. They grew in teaching skills such as engaging students to maximise their learning, giving guidance to students when they seemed lost, correcting students during their training, giving constructive feedback to students, and sharing their knowledge of community and disability resources. They described their openness to questions from the students and their willingness to share their perspectives as patients and educators. Many in Type B also expressed a desire for longer engagement periods, believing extended time would generate deeper attitudinal change in the medical students.

Although Type B patient educators held personal interests in advocacy, they intentionally did not allow advocacy aims to overshadow educational goals. As an educator with a disability, these patient educators perceived their primary role as easing future doctors into clinical practice, rather than emphasising disability rights.

### 3.5 Type C: Patient = Educator = Advocate

Patient, Educator, and Advocate roles are equally important

This type of patient educator described fully balancing all three identities. These individuals consistently viewed the roles of patient, educator, and advocate as equally vital and deeply interconnected. Their teaching combined personal narrative, explicit skill‐building for inclusive practice, and an emphasis on societal responsibilities toward persons with disabilities. The breadth of their sharing offered comprehensive exposure for learners, spanning micro‐level clinical interactions to macro‐level policy and inclusion issues.

Balancing all three roles provided them with the ability to give realistic perspectives on how PWD think and teach practical skills on how to help PWD, for example, relevant resources for PWD in the community and practical ways for society to be more inclusive.

They had also given thought to the kind of advocacy they brought to the workshop and focused on constructive and affirming suggestions to better society's awareness and understanding of PWD.

### 3.6 Identity D: Patient = Advocate > Educator

*Patient and Advocate roles are equally important with less emphasis on the Educator role*

Type D patient educators entered the curriculum with substantial prior advocacy leadership. They viewed the educator role as a platform for amplifying awareness of disability rights, structural barriers, and community needs. Their teaching frequently prioritised the collective interests of the disability community over clinical learning objectives.

Although students benefited from exposure to socio‐political realities of disability, there were occasions when messaging drifted away from the workshop's goal of building foundational clinical skills. These patient educators also voiced a need to change the curriculum to better suit their advocacy agenda, instead of aligning their message with the workshop's learning objectives. On other occasions, they described conveying inaccurate or incomplete views of healthcare, or use the platform to air unresolved grievances about the healthcare system.

## 4 Discussion

This study elucidated the possibility of patient educators with disabilities in a medical school curriculum having multifaceted roles, suggesting four patterns of engagement. Burke and Stets' Identity Theory may offer a useful framework to inform this context, as it posits that individuals hold multiple identities organised in a salience hierarchy, with the most prominent identity being the one most closely tied to the individual's core values [19]. Consistently with Identity Theory, our findings suggest that patient educators simultaneously held patient, educator, and advocate identities, with the relative salience of these identities shaping their teaching priorities, interactions with students, and alignment with curricular objectives.

Type A patient educators identified almost exclusively as patients with lived experience, often struggling with aspects of the educator role, particularly when role‐play diverged from their lived experience. Type B patient educators positively contributed to the learning experience through high teaching effectiveness, active engagement, balance between experience‐sharing and instructional needs. Type C patient educators positively contributed to holistic learning, sustained student engagement, with an additional element of disability empowerment.

Literature supports that simulated patients who work in medical school are to be carefully selected, well trained and competent to assess interviewing skills, patient education, and counselling skills [20]. Real patients selected to work in medical schools should ideally have good communication, good understanding of their clinical conditions, and be committed to education [2]. They should be able to deliver formative feedback during teaching sessions and be willing to share experiences in healthcare or personal aspects of their lives in teaching sessions [21]. These conclusions suggest that the PWD patient educators in Types B and C in our study had potentially the best predisposition of the four types to teach within this teaching context, although different identity configurations may offer distinct educational strengths. Type A patient educators may appear to benefit from support in the form of clear guidance, facilitation, and pedagogical training. Type D patient educators potentially contributed by increasing student awareness of disability justice and community contexts. This may be positive when students are empowered to advocate for patients, especially vulnerable populations [21, 22]. However, there is also the probable risk of overshadowing practical educational goals with advocacy priorities, which speaks in favour of delineating this individual type early on during the recruitment and initial training of PWD educators [23]. Patient educators should ideally be passionate about both medical education and improving the quality of health care delivery, without being overly bitter against the medical profession, using the teaching workshop to air their grievances, or solely advocating for inclusion [2, 20, 24]. This highlights the importance of careful selection and training of patient educators to ensure that their objectives broadly align with the classroom setting. It also highlights the critical role of having more experienced tutors serving as session facilitators alongside patient educators. These session facilitator tutors can guide discussions, including refocusing the session when necessary, and help patient educators balance personal advocacy with structured educational goals.

Our findings have potentially useful implications for the recruitment, selection, and training of all patient educators in medical education, not just PWD patient educators. During patient educator training, programmes could help patient educators recognise and articulate their intersecting identities, namely patient, educator, advocate, and any others, which may help them understand and, if possible, align their own expectations to the programme's with respect to their contributions and involvement. There is value in helping patient educators reflect on and understand their teaching identities including how to intentionally shift between roles as needed [25].

While our study only collected data at a single point in time, it may be possible that patient educators' perspectives on their roles and relative importance of these roles change over time. Rather than viewing patient educators as naturally belonging to one educator type and selecting them based on presumed types, programmes may also benefit from recognising that educator identity can develop over time. Structured opportunities for reflection, mentoring, observation, and feedback4 [24], may strengthen educator identity while helping all patient educators integrate advocacy and lived experience within educational goals.

While previous studies had highlighted the powerful learning experiences students gain from engaging with patient educators with disability [1, 2, 3, 5, 9, 11, 26], next steps in advancing this literature would be research on how different educator types impact student outcomes. Future research should also investigate whether the identity of PWD educators remains stable over time or tends to evolve after a longer period of exposure to the workshop and other educators. Such research would guide curriculum developers in refining their recruitment criteria and improving training processes to ensure both educational quality and inclusivity.

The other consideration will be studying the type of patient educators who are suitable in the democratisation of medical education whereby the patient educators are involved in all aspects of teaching and actively involved in all levels of decision making [27]. Such research will guide curriculum developers in co‐creation of courses. The emphasis on co‐creation was important as a course teaching about patients with disabilities without involving PWD or any other focused patient group risks being tokenistic [28].

## 5 Limitations

Given that participants were contributing to an institutional initiative and knew their feedback might inform future programme development, there is a possibility of social desirability bias in how they described their experiences, their roles, or perceived effectiveness. Although the study team ensured that the interviewer was a researcher who had not encountered the participants before, study participants were aware that the study team included tutors facilitating the workshop and the overseer of the programme recruiting patient educators.

While the study sought to explore diverse perspectives, the sample may not have been adequate to fully capture the range of experiences across different disability types, teaching experiences, or levels of comfort with role‐play and teaching. Although all patient educators were invited to participate in the study, only half of the current patient educators took part. Our recruitment strategy did not allow to characterise the profiles of non‐participants, nor their reasons for declining to participate. Because of the limitation in sample size and participation, we could not achieve thematic saturation. This limits our findings from capturing the entirety of patient educators' experiences and the certainty with which we can defend the exhaustiveness of the four types of educators we identified in this study. This programme also only included patient educators with specific types of disabilities (hearing loss, vision impairment, and mobility limitations). Patient educators with other types of disabilities may have different perspectives and be different types of educators. Research with more PWD educators in similar contexts will be beneficial.

## 6 Conclusions

Patient educators with physical disabilities contribute to medical education through interrelated identities as patients, educators, and advocates. These roles are fluid and variably emphasised, influencing teaching style and effectiveness. Understanding these role configurations can help institutions design supportive frameworks that strengthen pedagogical competence while preserving authenticity of lived experience. Recruitment should prioritise individuals with an interest in teaching, while training should incorporate guidance on educational delivery, role balance, and constructive feedback. Faculty facilitation remains crucial in ensuring alignment between patient educators' contributions and curricular objectives. Cultivating this balance fosters both inclusive learning environments and deeper professional identity formation among medical students.

## Author Contributions

**Vivien Min Er Lee:** conceptualisation, investigation, writing – original draft, methodology, validation, visualisation, writing – review and editing, formal analysis, project administration, data curation. **Laurie J. Goldsmith:** investigation, methodology, validation, visualisation, writing – review and editing, formal analysis, supervision, data curation. **Anna Szücs:** conceptualisation, investigation, methodology, validation, visualisation, writing – review and editing, formal analysis, data curation, supervision. **V Vien Lee:** investigation, methodology, validation, visualisation, writing – review and editing, formal analysis, data curation. **Jun Cong Goh:** conceptualisation, investigation, project administration, data curation, formal analysis, methodology, validation, visualisation. **Jeffrey Jiang:** conceptualisation, investigation, methodology, validation, writing – review and editing, formal analysis, data curation, visualisation. **Nicola Ngiam:** conceptualisation, methodology, validation, writing – review and editing, formal analysis, data curation, supervision, visualisation. **José M. Valderas:** conceptualisation, methodology, validation, visualisation, writing – review and editing, formal analysis, data curation, supervision, resources. **Victor Weng Keong Loh:** conceptualisation, investigation, methodology, validation, visualisation, writing – review and editing, formal analysis, data curation, supervision, resources.

## Ethics Statement

All procedures were approved by the Institutional Review Board of the National University of Singapore (NUS‐IRB‐2023‐154).

## Conflicts of Interest

Authors VLME, JJ and VWKL are tutors in the “Communications with Persons with Disabilities” workshop. NN oversees the programme that manages the recruitment and training of the PWD patient educators. There are otherwise no conflicts of interest.

## Supporting information


Supporting File

## Data Availability

Data is available on request from the corresponding author.
