# Supplementary material for: “Engagement Is Not One‐Dimensional”: The Multiple Roles of Patient Educators With Physical Disabilities in Family Medicine
Source: Health Expect. 2026 Aug 26;29(5):e70851. doi: 10.1111/hex.70851 (PMC13518373; doi:10.1111/hex.70851)
Supplement: Supplementary file 1 [file HEX-29-e70851-s001.docx]

**Supplementary Materials**

**COREQ Checklist**

**Patient Educator Interview Topic Guide**

A. Explore their **experiences** as patient educators [20 minutes]

- How was your experience in teaching in this curriculum?
- How did you feel when you were participating in this curriculum?

(Any anxieties, surprises or difficulties faced?)

- How did you feel about working with medical students?

(Any highlights? Any frustrations? Any memorable examples?)

- How did you feel about working with tutors?

(Any highlights? Any frustrations? Any memorable examples?)

- Could you tell us why did you choose to be a patient educator?
- What do you think are the benefits of your involvement in this workshop for medical students?
- What tips would you give to future patient educators?

B. Explore the perspectives of patient educators toward how the PWD curriculum may affect students’ **humanistic medical professional identity** [20 minutes]

- Share with me your perspective of students’ attitudes towards disability when you interacted with them.
- Do you think there would be any change in the students’ knowledge, skills, and attitudes before and after the workshop?
- Are there any parts of the curriculum that seemed to be particularly impactful/remarkable to the students? (Any examples?)
- Do you think this curriculum might affect the students in the long term? If so, how might they be affected?
- Do you think this curriculum might affect the students’ humanistic values / values as future doctors in the long term? If so, how?

C. Explore the **strengths and weaknesses** of the workshop [15 minutes]

- Was there any aspect of the curriculum that was particularly impactful/remarkable for you as a patient educator? (How was it beneficial, and were there any detriments?)
- How could this curriculum be more impactful in shaping the values of students as future doctors?
- How do you think the curriculum for providing healthcare for persons with disability could be improved?
- Do you feel that the curriculum should be extended to doctors and other healthcare professionals?
- Is there anything else you think we should include in the teaching of medical students on helping persons with disabilities?

D. **Concluding** the interview [5 minutes]

- How was the interview?
- Is there anything else that you would like to share?

**Patient Educator Interview Topic Guide (Iterative)**

**(*Patient educators as EDUCATORS) WHY is their voice important in medical education?)**

A. Explore their **experiences** as patient educators [45 minutes]

- Tell me about your disability
- When did you start teaching in this workshop? How many times previously? Currently still teaching/a patient educator? If no, why?
- Why did you decide to sign up to be a patient educator?
- How would you describe your role as a patient educator?
- What do you think about the role of patient educators in medical education? How valuable do you think it is to have patient educators in medical education?
- How was your experience in teaching in this curriculum?
- How did you feel when you were participating in this curriculum?

*(Any anxieties, surprises or difficulties faced?)*

- How did you feel about working with medical students? Student’s attitudes towards PWD? What do you hope students will get out of this? Any misunderstandings/discrepancy in knowledge? What did you have to explain to them?

*(Any highlights? Any frustrations? Any memorable examples?)*

- How did you feel about working with tutors? Any misunderstandings/discrepancy in knowledge? What did you have to explain to them?
- *(Any highlights? Any frustrations? Any memorable examples?)*
- Was there any aspect of the curriculum that was particularly impactful/remarkable for you as a patient educator? (How was it beneficial, and were there any detriments?)
- How do you think you have grown as a patient educator?
- What you have learnt being a patient educator?
- What do you think are the benefits of your involvement in this workshop for medical students?
- What do you think you will do to be a better patient educator?
- What tips would you give to another PWD who would like to be a patient educator?

B. Explore the perspectives of patient educators toward how the PWD curriculum may affect students’ **humanistic medical professional identity** [20 minutes]

- Share with me your perspective of students’ attitudes towards disability when you interacted with them.
- Do you think there would be any change in the students’ knowledge, skills, and attitudes before and after the workshop?
- Are there any parts of the curriculum that seemed to be particularly impactful/remarkable to the students? (Any examples?)
- Do you think this curriculum might affect the students in the long term? If so, how might they be affected?
- Do you think this curriculum might affect the students’ humanistic values / values as future doctors in the long term? If so, how?

C. Explore the **strengths and weaknesses** of the workshop [15 minutes]

- How could this curriculum be more impactful in shaping the values of students as future doctors?
- How do you think the curriculum for providing healthcare for persons with disability could be improved?
- Do you feel that the curriculum should be extended to doctors and other healthcare professionals?
- Is there anything else you think we should include in the teaching of medical students on helping persons with disabilities?

D. **Concluding** the interview [5 minutes]

- How was the interview?
- Is there anything else that you would like to share?
